# mPPTMP195 nanoparticles enhance fracture recovery through HDAC4 nuclear translocation inhibition

**DOI:** 10.1186/s12951-024-02436-1

**Published:** 2024-05-17

**Authors:** Xinping Chen, Chengwei Li, Jiyu Zhao, Yunxiang Liu, Zhizhong Zhao, Zhenyu Wang, Yue Li, Yunfei Wang, Lixia Guo, Lu Li, Chongwei Chen, Bing Bai, Shaowei Wang

**Affiliations:** 1 Shanxi Key Lab of Bone and Soft Tissue Injury Repair, Department of Orthopedics, The Second Hospital of Shanxi Medical University, Taiyuan, PR China; 2 Department of Biochemistry, Shanxi Medical University, Basic Medical College, Taiyuan, 030001 PR China; 3 Department of Surgery, Tongji Shanxi Hospital, Shanxi Bethune Hospital, Shanxi Academy of Medical Science, Third Hospital of Shanxi Medical University, Taiyuan, 030032 PR China; 4 School of Pharmacy, Shanxi Medical University, Taiyuan, 030001 PR China; 5 Key Laboratory of Bioorganic Phosphorus Chemistry & Chemical Biology, Department of Chemistry, Tsinghua University, Beijing, 100084 PR China

**Keywords:** Fracture, HDAC4, NRF2/HO-1 signaling pathway, Osteoblast, Osteoclast

## Abstract

Delayed repair of fractures seriously impacts patients’ health and significantly increases financial burdens. Consequently, there is a growing clinical demand for effective fracture treatment. While current materials used for fracture repair have partially addressed bone integrity issues, they still possess limitations. These challenges include issues associated with autologous material donor sites, intricate preparation procedures for artificial biomaterials, suboptimal biocompatibility, and extended degradation cycles, all of which are detrimental to bone regeneration. Hence, there is an urgent need to design a novel material with a straightforward preparation method that can substantially enhance bone regeneration. In this context, we developed a novel nanoparticle, mPPTMP195, to enhance the bioavailability of TMP195 for fracture treatment. Our results demonstrate that mPPTMP195 effectively promotes the differentiation of bone marrow mesenchymal stem cells into osteoblasts while inhibiting the differentiation of bone marrow mononuclear macrophages into osteoclasts. Moreover, in a mouse femur fracture model, mPPTMP195 nanoparticles exhibited superior therapeutic effects compared to free TMP195. Ultimately, our study highlights that mPPTMP195 accelerates fracture repair by preventing HDAC4 translocation from the cytoplasm to the nucleus, thereby activating the NRF2/HO-1 signaling pathway. In conclusion, our study not only proposes a new strategy for fracture treatment but also provides an efficient nano-delivery system for the widespread application of TMP195 in various other diseases.

**Supplementary Information:**

The online version contains supplementary material available at 10.1186/s12951-024-02436-1.

## Background

During a fracture, osteoclasts are activated and initiate the resorption of necrotic bone tissue. This resorption exposes and facilitates the regeneration of the surrounding healthy bone tissue. Subsequently, osteoblasts are attracted to the fracture site and commence the synthesis of new bone matrix after the osteoclasts remove the necrotic bone tissue [1–3]. These two types of cells collaborate to ultimately restore the structure and function of the bone.

HDAC4, an IIa histone deacetylase, plays a pivotal role in the transcriptional regulation of genes within the nucleus and is essential in various cell types. Within the skeletal system, HDAC4 is critical for bone growth, remodeling, and repair. It orchestrates the differentiation of bone marrow stromal cells (BMSCs) into osteoblasts, thus facilitating the growth and repair of bone tissue.

TMP195 belongs to the novel class IIa HDAC inhibitors and interacts with zinc ions situated at the base of the class IIa HDAC catalytic pocket, functioning as a specific inhibitor of HDAC4 [4]. It has demonstrated effectiveness in treating various conditions, including atherosclerosis [5], cardiomyocyte hypertrophy [6–8], acute kidney injury [9], chronic lymphocytic leukemia [10], tuberculosis [11], and tumor-related diseases like breast cancer [12–16]. Nonetheless, the application of TMP195 in regulating osteoclast differentiation and bone marrow mesenchymal stem cell differentiation into osteoblasts for fracture repair has not been previously documented. This research aimed to explore the effect of TMP195 on osteoclast and bone marrow mesenchymal stem cell differentiation for fracture repair. Regrettably, the main limiting factors for the application of TMP195 are its low solubility in biological fluids and inadequate bioavailability for topical administration.

PEG is a widely utilized hydrophilic polymer renowned for its excellent biocompatibility and enhanced permeation retention; it has received FDA approval for medical applications [17]. PCL, a commonly used hydrophobic block, finds widespread use in various drug delivery systems and is also FDA-approved for medical applications [18].

Utilizing the aforementioned advantages, our study focused on fabricating polymeric micelles using mPEG-PCL as the foundation to encapsulate TMP195, denoted as mPPTMP195, aiming to achieve controlled and sustained drug release, thereby enhancing the drug’s bioavailability in vivo. The outcomes presented in this paper offer an efficient nano-delivery system, enhancing the fracture repair capabilities of TMP195 and fostering its potential application in various other diseases.

## Results

### Synthesis and characterization of mPEG-PCL and mPPTMP195

**Fig. 1:**
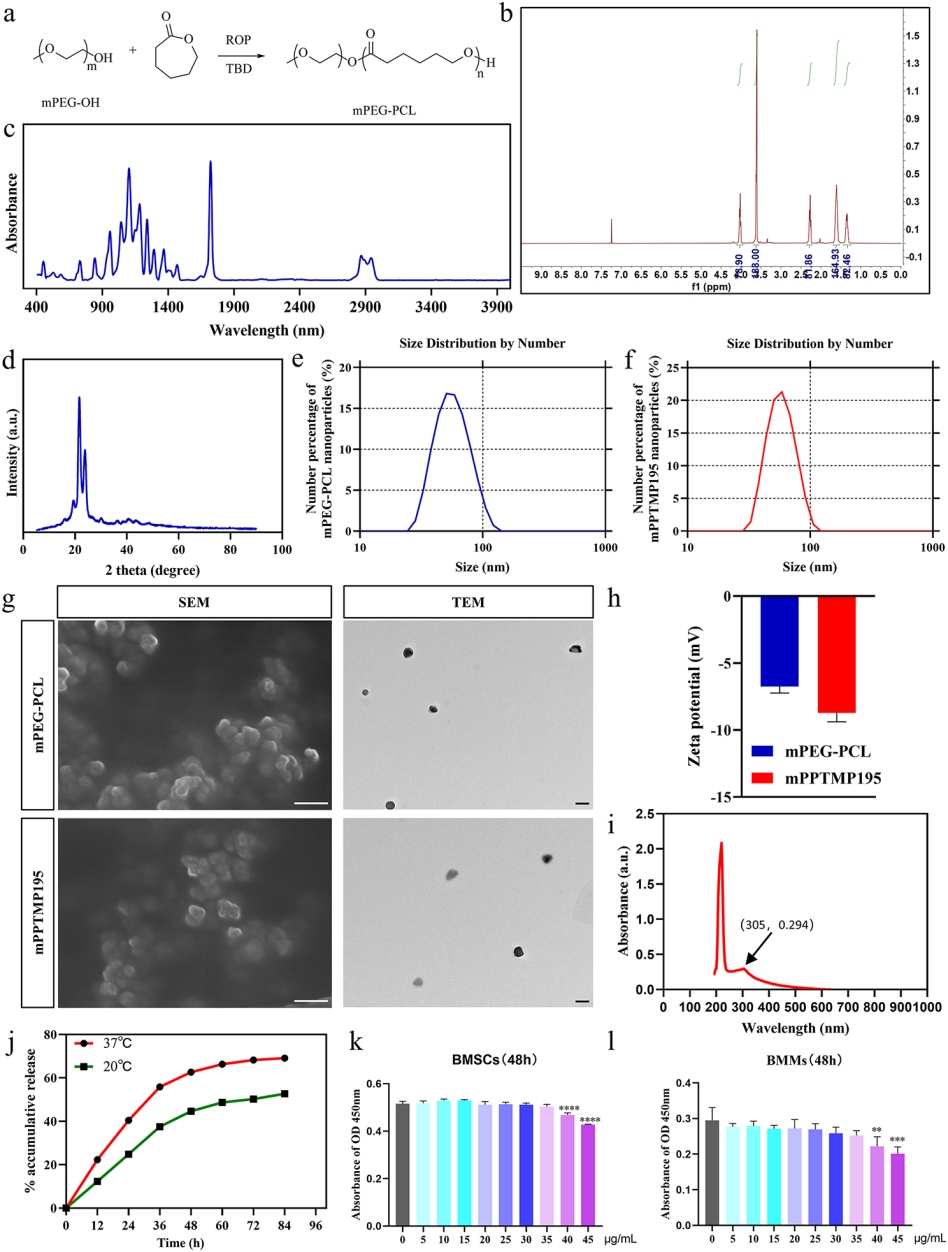
Characterization of mPPTMP195 nanomaterials. (**a**) Synthesis roadmap of mPEG-PCL. (**b**) 1 H NMR spectrum of mPEG-PCL. (**c**) FTIR spectrum of mPEG-PCL. (**d**) XRD pattern of mPEG-PCL. (**e**) DLS analysis of mPEG-PCL. (**f**) DLS profile of mPPTMP195. (**g**) SEM and TEM images of mPEG-PCL and mPPTMP195. (**h**) Zeta potential of mPEG-PCL and mPPTMP195. (**i**) Characteristic absorption peaks of TMP195 detected by UV spectrophotometer. (**j**) Retardation curves of mPPTMP195 at different temperatures. (**k**) CCK-8 assay to assess the toxicity of mPPTMP195 to BMSCs at different concentrations. (**l**) CCK-8 assay to assess the toxicity of different concentrations of mPPTMP195 to BMMs. All bars are expressed as mean ± SD. ^**^P < 0.01, ^***^P < 0.001 and ^****^P < 0.0001relative to the control group. Differences were analyzed by Student’s t-test

Firstly, we prepared mPEG-PCL nanoparticles, as shown in Fig. 1a. By 1 H NMR mapping, a strong peak in the region of 3.5-4.0 ppm was observed, corresponding to the methylene hydrogen atom (-OCH2-) of mPEG. The chemical shift in this region is typical, and the single signal indicates a high degree of reproducibility of this part of the structure. Several peaks in the 2.0-2.5 ppm region can be attributed to the hydrocarbon alkane region of the PCL, with a bias towards peaks with a δ of about 2.3 (e.g., δ 2.31), likely corresponding to the methylidene hydrogen atoms near the carbonyl group in the PCL unit (-COCH2-). Additionally, several peaks can be observed in the region of 1.0–2.0 ppm, attributing signals to other methylene hydrogen atoms in the PCL part. A small signal may be observed at δ about 3.6, which is more difficult to distinguish and may correspond to the trailing methylene hydrogen atoms of the PCL or to some structure at the mPEG-PCL switching point. In the high-field region, especially where δ is below 1.5 ppm, the signal is attributed to the hydrogen atom of the methylene group on the PCL chain. Overall, the observed hydrogen NMR spectra were compatible with the expected mPEG-PCL structure: the methylene-associated peaks of mPEG were at 3.5-4.0 ppm, and the methylene-hydrogen-associated peaks of PCL were at 1.0-2.5 ppm. This supports the synthesis of the mPEG-PCL copolymer (Fig. 1b).

Then, by FTIR analysis, sharp peaks appearing near 2900 nm were found, corresponding to the stretching vibration of the alkyl-C-H bond, indicating the presence of alkyl chains. This is consistent with the fact that both mPEG and PCL have an alkyl portion. The sharp peak near 1750–1800 nm corresponds to the stretching vibration of the ester bond (C = O), a characteristic strong absorption peak in PCL, indicating the presence of a PCL component. The absorption peak in the interval of 1000–1300 nm corresponds to a probable C-O-C stretching vibration, pointing to an ether bond in the mPEG fraction. The pattern of absorption peaks shown in the FTIR pattern is consistent with the expected absorption peaks of ester and ether functional groups in the mPEG-PCL polymer, indicating successful synthesis of mPEG-PCL (Fig. 1c).

Based on the provided XRD (X-ray diffraction) pattern, a very sharp and intense diffraction peak was observed at lower 2theta values (around 24 degrees or so), indicating a pronounced crystallinity of the copolymer. This peak may correspond to the characteristic crystal structure of PCL. PCL is recognized as a polyester with semi-crystalline nature, and its crystalline region causes sharp diffraction peaks to appear on the XRD pattern (Fig. 1d). These results indicate the successful preparation of mPEG-PCL.

Next, we encapsulated TMP195 using mPEG-PCL nanoparticles. From the DLS patterns, the average particle size of mPEG-PCL was found to be 57.88 ± 18.7 nm (Fig. 1e), and the average particle size of mPPTMP195 was 59.3 ± 15.0 nm (Fig. 1f), indicating little change in the particle size of mPEG-PCL after loading TMP195. Observed by SEM and TEM, mPPTMP195 was spherical, with a regular structure, smooth surface, and uniform distribution. There was little difference in the diameter size between mPPTMP195 and mPEG-PCL nanoparticles (Fig. 1g). By zeta potential, it was found that the zeta potential of mPEG-PCL was about − 10 mV, and the absolute value of the zeta potential of mPPTMP195 was slightly lower, about − 5 mV, which may indicate that mPEG-PCL may be more prone to aggregation than mPPTMP195 in suspension under the same conditions, but this difference was not particularly significant (Fig. 1h).

Finally, the characteristic UV absorption peak of TMP195 appeared at 305 nm using a UV spectrophotometer (Fig. 1i). Therefore, the detection wavelength of TMP195 was set at 305 nm. By measuring the UV absorbance at 305 nm for different concentrations of mPPTMP195, repeating three times and taking the average value to plot the standard curve, mPPTMP195 exhibited sustained release (Supplementary Fig. 1a). Based on the standard curve, the release properties of mPPTMP195 at different temperatures of its contained TMP195 were determined. It was found that the release of TMP195 was faster at 37 °C compared to 20 °C, with about 70% released in 72 h (Fig. 1j), satisfying effective sustained release in organisms. To assess the cytotoxicity of mPPTMP195, it was found that mPPTMP195 in the concentration range of 0–35 µg/mL had no biotoxic effect either in BMSCs or in BMMs (Fig. 1k and l).

### mPPTMP195 nanoparticles promote differentiation of BMSCs to osteoblasts

**Fig. 2:**
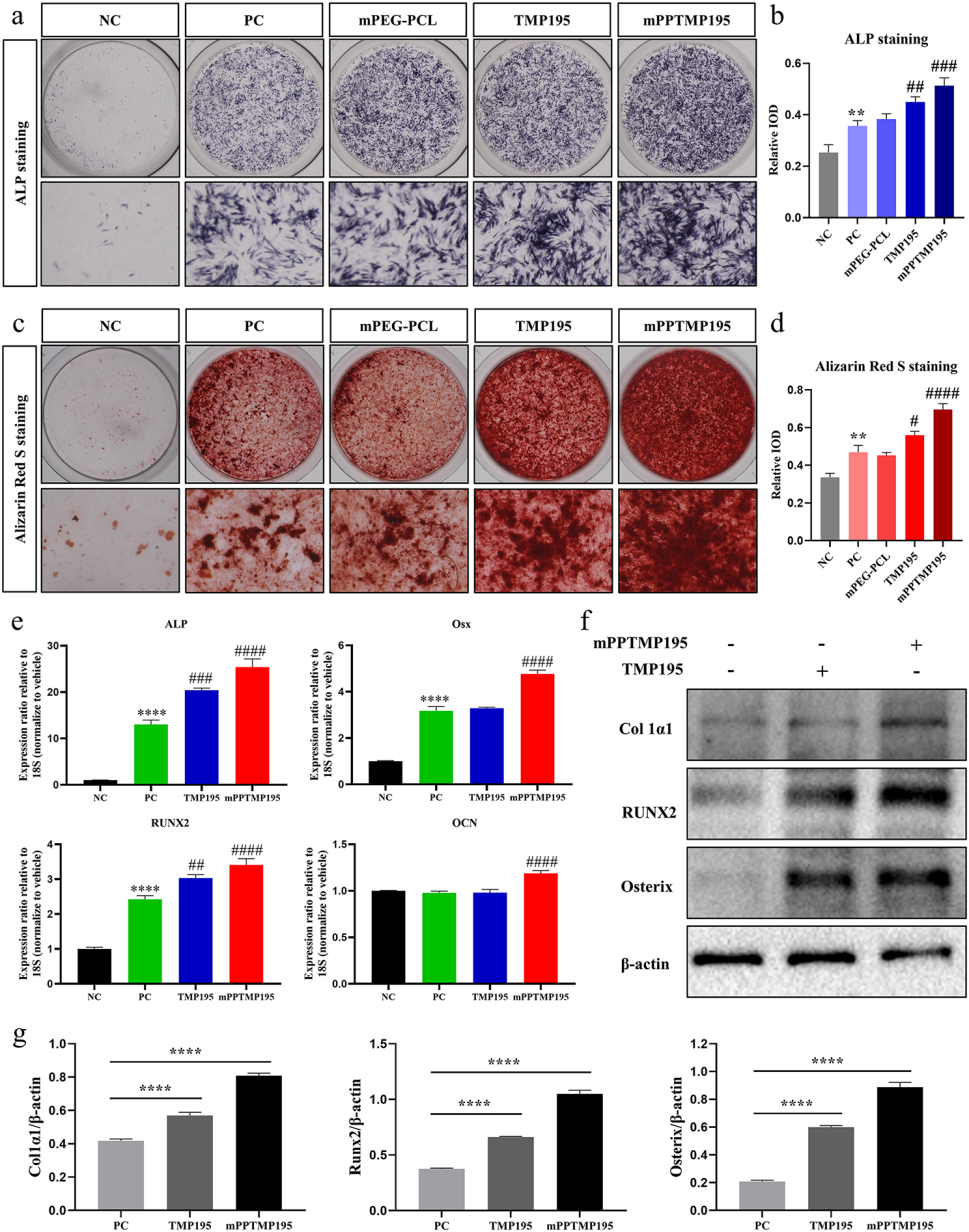
mPPTMP195 promotes the osteoblastic differentiation of BMSCs. (**a**) mPPTMP195 treatment induced ALP staining in BMSCs after 7 days. (**b**) Quantification of ALP-positive cells. (**c**) mPPTMP195 treatment induced alizarin red staining of BMSCs after 14 days. (**d**) Quantification of alizarin red staining into bone mineralization. (**e**) Assessment of ALP, Osx, RUNX2, and OCN gene expression in mPPTMP195-treated BMSCs undergoing osteogenic differentiation. (**f**) Western blot analysis evaluating the expression of Col1α1, RUNX2, and Osterix in BMSCs following 7 days of mPPTMP195 treatment. β-actin served as the internal reference protein. (**g**) Quantification of gray values of Western blot bands. ^**^P < 0.01 and ^****^P < 0.0001 indicate comparison with the NC group. ^#^*P* < 0.05, ^##^*P* < 0.01, ^###^*P* < 0.001 and ^####^*P* < 0.0001 indicates compared to the PC group

To assess the osteogenesis-promoting effect of mPPTMP195 nanoparticles on bone marrow MSCs differentiation into osteoblasts, we initially cultured primary bone marrow MSCs for osteogenic induction. Positive alkaline phosphatase staining (ALP) or alizarin red staining (AR) on day 7 and day 14 of induction in the positive control group (PC group) confirmed successful osteoblast differentiation. In contrast, the negative control group (NC group) did not exhibit these staining patterns, indicating successful induction of BMSCs differentiation into osteoblasts. Notably, the addition of mPEG-PCL did not affect ALP and AR staining, suggesting its non-toxic effect on BMSCs osteogenesis. Moreover, both the free TMP195 and mPPTMP195 nanoparticle groups showed darker staining compared to the NC group, indicating a higher number of positive cells. However, the mPPTMP195 nanoparticle group exhibited more mineralized nodules than the other groups (Fig. 2a, c). Quantitative analysis revealed higher optical density of cellular staining for ALP and AR in the mPPTMP195 group compared to the other groups, with ALP staining and AR staining in the mPPTMP195 nanoparticle group being approximately 1.2-fold and 1.3-fold higher than the free TMP195 group, respectively (Fig. 2b, d). This suggests that mPPTMP195 has a superior osteogenesis promotion effect compared to the free TMP195 group. Next, we measured the content of TMP195 in the culture medium using HPLC and calculated the degradation rate of TMP195, which indirectly reflected the uptake rate of TMP195 by BMSCs. We observed no significant difference in the degradation rate of TMP195 between the free TMP195 group and the mPPTMP195 group. However, regarding cellular uptake, TMP195 was more readily taken up by cells in the mPPTMP195 group (Supplementary Fig. 1b). Therefore, the mPPTMP195 group exhibited a superior osteogenesis-promoting effect compared to the free TMP195 group.

Subsequently, we assessed the expression of transcription factors essential for osteoblast formation by free TMP195 and mPPTMP195 nanoparticles through RT-PCR, including ALP, Osx, RUNX2, and OCN. Upregulation of all these genes in the PC group compared to the NC group confirmed BMSCs differentiation towards osteoblasts. Interestingly, ALP and RUNX2 genes were upregulated in the free TMP195 group compared to the PC group, while there was no difference in the expression of Osx and OCN genes. However, in the mPPTMP195 nanoparticle group, the expression of all four genes was up-regulated compared to the PC group, with a more pronounced upregulation than in the free TMP195 group (Fig. 2e). These findings suggest that mPPTMP195 nanoparticles may exhibit higher stability than free TMP195 in promoting BMSCs differentiation into osteoblasts.

Finally, we assessed the expression of key proteins involved in osteoblast differentiation through Western blot analysis. Both free TMP195 and mPPTMP195 nanoparticle-treated BMSCs showed significantly increased expression of Col1α1, RUNX2, and Osterix, key proteins in osteoblast differentiation. However, this effect was more pronounced in the mPPTMP195 group (Fig. 2f). Quantitative analysis revealed that mPPTMP195 nanoparticles induced approximately 1.3-fold higher expression of Col1α1, 1.7-fold higher expression of RUNX2, and 1.5-fold higher expression of Osterix than free TMP195 (Fig. 2g). These results suggest that mPPTMP195 promotes BMSCs differentiation into osteoblasts more effectively than free TMP195.

### mPPTMP195 nanoparticles inhibit differentiation of BMMs to osteoclasts

**Fig. 3:**
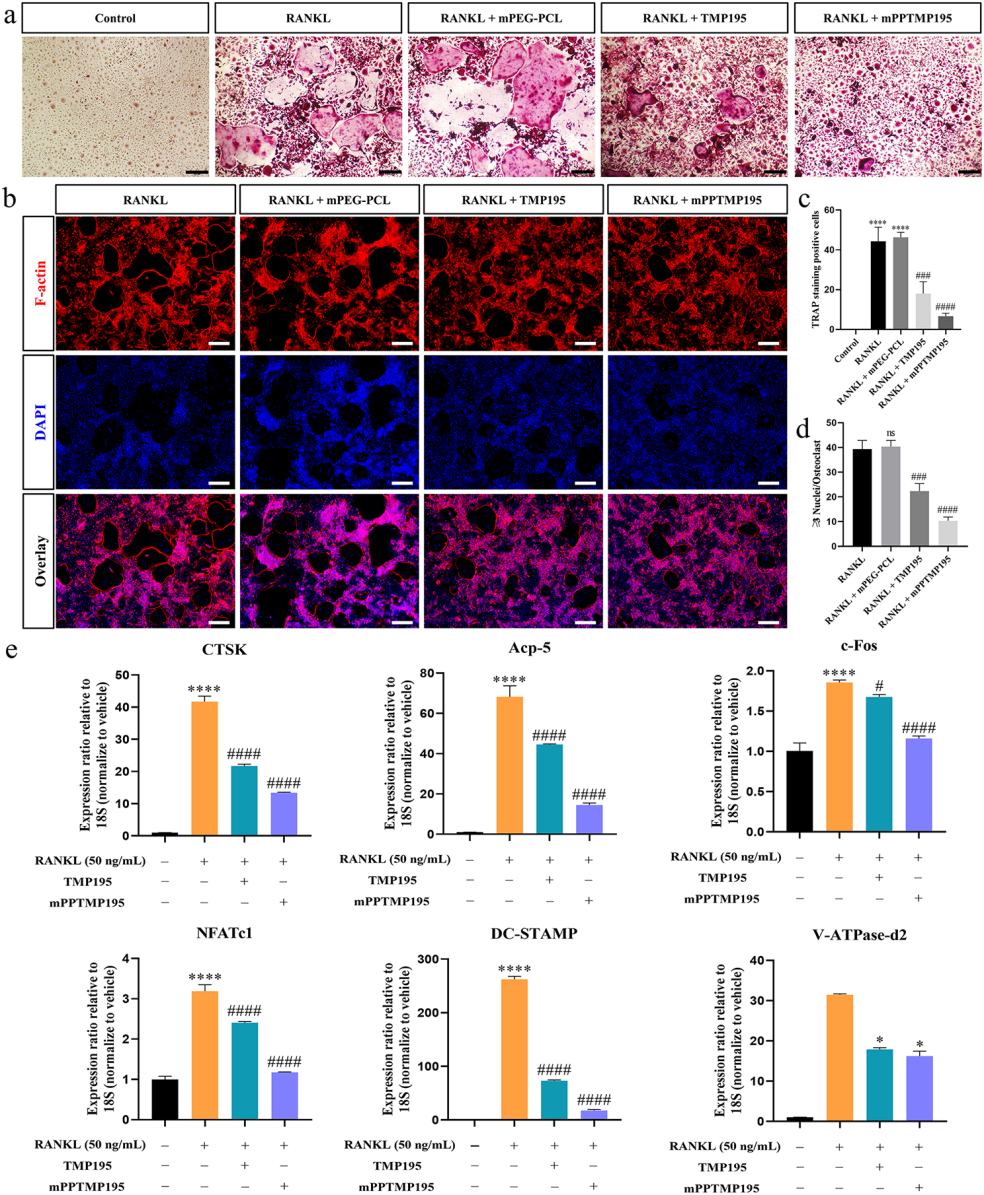
mPPTMP195 inhibits the differentiation of BMMs into osteoclasts. (**a**) TRAP staining of osteoclasts. Black scale bar: 200 μm. (**b**) Cytoskeletal fluorescence staining image of osteoclasts (nucleus in blue, cytoskeleton in red). White scale bar: 200 μm. (**c**) Quantification of TRAP staining-positive osteoclasts. (**d**) Quantification of cytofluorescence staining. (**e**) Expression of osteoclast-specific mRNAs, including CTSK, Acp-5, c-Fos, NFATc1, DC-STAMP, and V-ATPase-d2, was analyzed by RT-PCR. All bars are expressed as mean ± SD. ^*^P < 0.05 and ^****^P < 0.0001 indicate comparison with the control group. ^#^*P* < 0.05, ^##^*P* < 0.01, ^###^*P* < 0.001 and ^####^*P* < 0.0001indicates compared to the RANKL group

To investigate the impact of mPPTMP195 nanoparticles on osteoclast differentiation, primary bone marrow mononuclear macrophages (BMMs) from mice were cultured for osteoclast induction. TRAP staining revealed a significant increase in positive osteoclasts compared to control and RANKL-induced cells, indicating successful differentiation of BMMs into osteoclasts. The addition of mPEG-PCL did not affect RANKL-induced osteoclast differentiation, indicating no interference with mPEG-PCL. Both free TMP195 and mPPTMP195 nanoparticles inhibited RANKL-induced osteoclast differentiation, with mPPTMP195 demonstrating greater inhibition compared to free TMP195 (Fig. 3a). Further staining with ghost-pen cyclic peptide showed reduced osteoclast fusion and lower cell count in the presence of free TMP195 or mPPTMP195 compared to RANKL-induced cells, with no impact observed with mPEG-PCL (Fig. 3b). Quantitative analysis revealed a significant reduction in the number of osteoclasts in the mPPTMP195 nanoparticle group compared to the other groups, approximately 2.7-fold lower than the free TMP195 group (Fig. 3c). Additionally, the number of osteoclasts in the mPPTMP195 nanoparticle group was about 2.2-fold lower than the free TMP195 group in quantitative analysis of ghost pen cyclic peptide staining (Fig. 3d). HPLC analysis of the induction medium confirmed that there was no significant difference in the degradation rate of TMP195 released from mPPTMP195 nanoparticles compared to free TMP195 drug in the medium. However, BMMs exhibited greater uptake of TMP195 in the mPPTMP195 group compared to the free TMP195 group over the same period of time (Supplementary Fig. 1c), a trend consistent with BMSCs differentiation during osteogenesis (Supplementary Fig. 1b).

We assessed the expression of genes associated with osteoclast formation (NFATc1 and c-Fos), fusion (DC-STAMP and V-ATPase-d2), and function (Acp5 and CTSK) to elucidate mPPTMP195’s effect on RANKL-induced osteoclast differentiation and function. RANKL stimulation upregulated NFATc1, c-Fos, DC-STAMP, Acp5, CTSK, and V-ATPase-d2 gene expression, indicating osteoclast differentiation. However, both free TMP195 and mPPTMP195 downregulated the expression of these genes induced by RANKL, with mPPTMP195 showing stronger inhibition compared to free TMP195 (Fig. 3e).

**Fig. 4:**
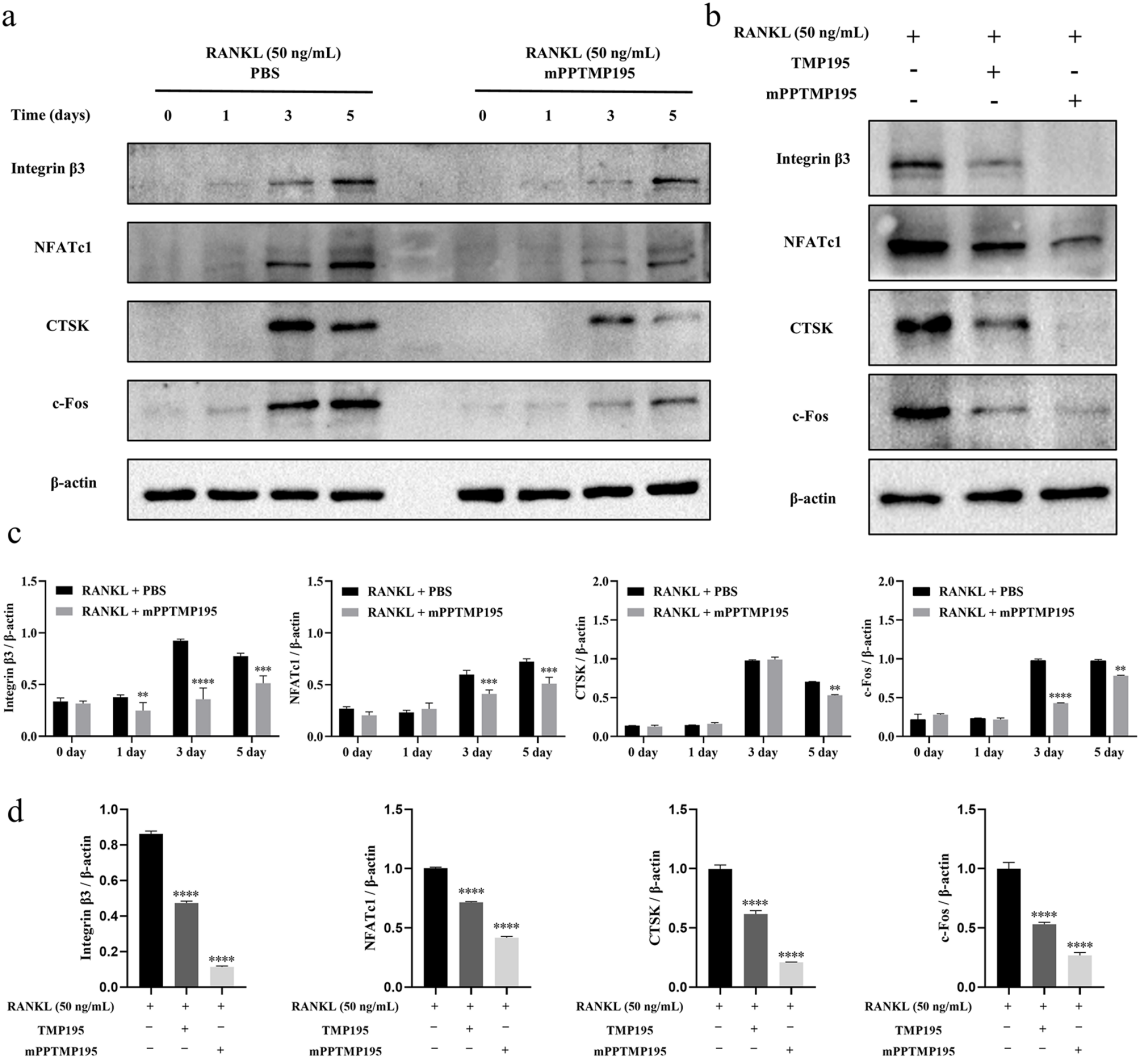
mPPTMP195 inhibits the expression of key proteins during the differentiation of BMMs into osteoclasts. (**a**) After 0, 1, 3, and 5 days of treatment with RANKL (50 ng/mL) in the presence or absence of mPPTMP195, Western blot analysis of Integrin β3, NFATc1, CTSK, and c-Fos expression was conducted. β-actin served as the internal reference protein. (**b**) Western blot analysis of Integrin β3, NFATc1, CTSK, and c-Fos expression in BMMs treated with RANKL (50 ng/mL) for 5 days in the presence or absence of mPPTMP195. β-actin was used as an internal reference protein. (**c**) and (**d**) Statistical analysis of the gray values of Western blot bands. All bars are expressed as mean ± SD. ^**^P < 0.01, ^***^P < 0.001 and ^****^P < 0.0001 indicates compared to the RANKL group

Western blotting showed a time-dependent increase in Integrin β3, NFATc1, CTSK, and c-Fos expression induced by RANKL. Treatment with mPPTMP195 nanoparticles inhibited RANKL-induced expression of proteins related to osteoclast formation and function, suggesting time-dependent inhibition of osteoclasts by mPPTMP195 nanoparticles (Fig. 4a). Comparison between free TMP195 and mPPTMP195 nanoparticle effects on osteoclast differentiation revealed both inhibited osteoclast formation and related proteins. Quantitative analysis showed mPPTMP195 nanoparticles inhibited Integrin β3 from day 1, NFATc1 and c-fos from day 3, and CTSK from day 5, with a significantly higher inhibition compared to free TMP195 (Fig. 4c, d). These results suggest that mPPTMP195 nanoparticles not only inhibit BMMs differentiation into osteoclasts but are also more effective than free TMP195.

### mPPTMP195 nanoparticles promote bone fracture healing

To assess the impact of mPPTMP195 nanoparticles on fractures in vivo, we established a femoral fracture model with intramedullary pin internal fixation in 12-week-old male C57BL/6 mice, simulating a stable fracture.

**Fig. 5:**
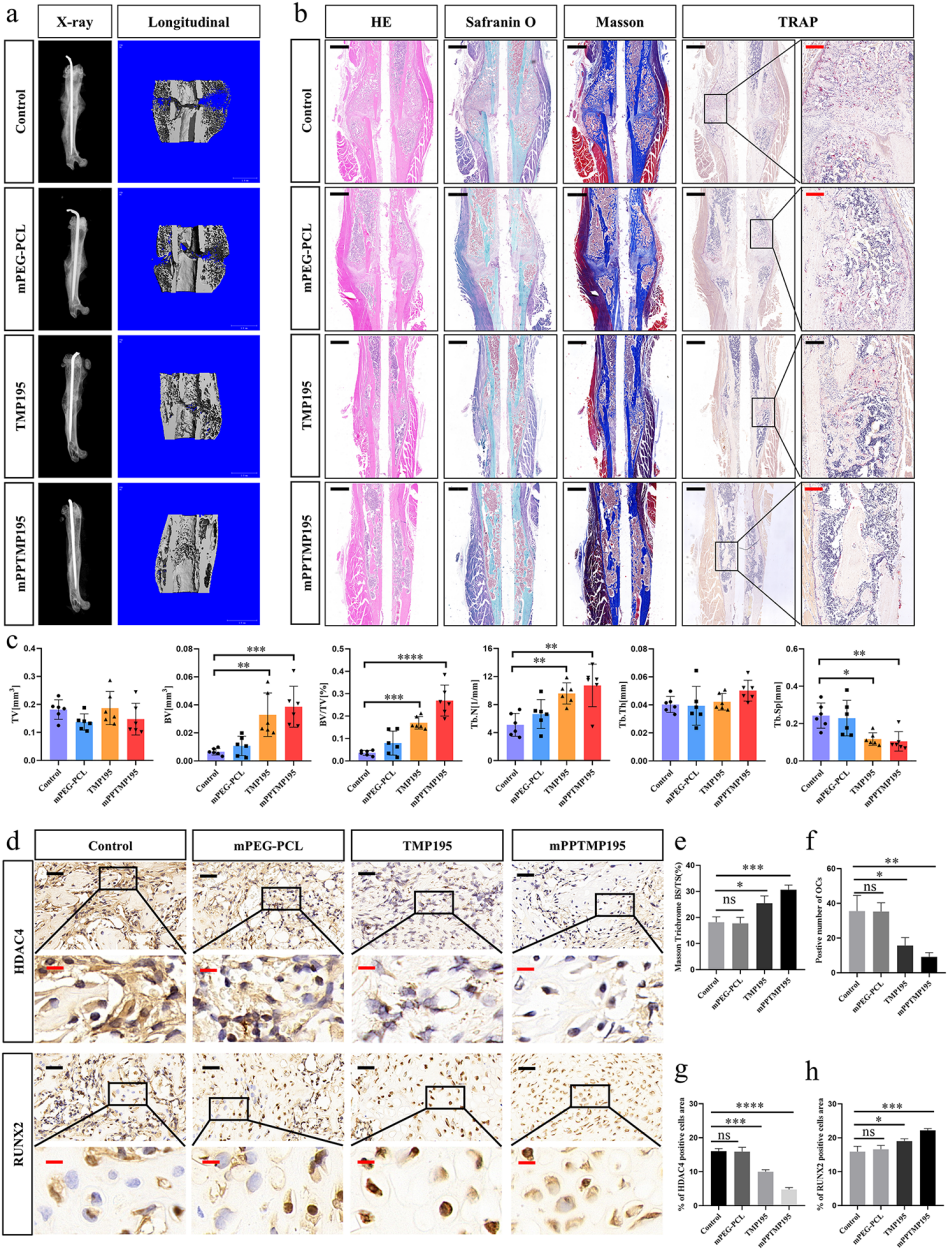
mPPTMP195 promotes fracture repair. (**a**) Representative three-dimensional micro-CT images of bone repair in control, mPEG-PCL, TMP195, and mPPTMP195 mice 14 days after fracture. (**b**) Hematoxylin-eosin, saffron O fast green, Masson’s trichrome staining, and TRAP staining at the bone scab. Black scale: 200 μm. Red scale: 40 μm. (**c**) Micro CT analysis of bone tissue data. (**d**) Immunohistochemical staining images of HDAC4 and RUNX2 at the bone scabs of control, mPEG-PCL, TMP195, and mPPTMP195 mice. Black scale: 40 μm. Red scale: 10 μm. (**e**) Masson’s trichrome staining was performed to quantify newly formed bone at the scab; BS/TS, bone surface/tissue surface. (**f**) TRAP trichrome staining was used to quantify positive osteoclasts at the bone scab. (**g**) Quantification of the area occupied by HDAC4-positive cells. (**h**) Quantification of the area occupied by RUNX2-positive cells. All bars are expressed as mean ± SD. ^*^P < 0.05, ^**^P < 0.01, ^***^P < 0.001 and ^****^P < 0.0001 relative to the control group. Differences were analyzed by Student’s t-test

Local subcutaneous injection of mPPTMP195 nanoparticles into the fracture region was performed, followed by Micro-CT scans and analysis after 2 weeks. CT tomography revealed no significant difference between the control and mPEG-PCL groups. However, mice injected with free TMP195 and mPPTMP195 nanoparticles exhibited a high-density signal near the fracture line, indicative of newly generated bone scab (Fig. 5a). Data analysis showed a slight increase in BV/TV in the free TMP195 group compared to the control group. Notably, the mPPTMP195 group displayed a significant increase in bone volume (BV), bone volume fraction (BV/TV), and trabecular number (Tb.N), along with a significant decrease in trabecular separation (Tb.Sp) compared to the other groups. Though tissue volume (TV) and trabecular thickness (Tb.Th) showed no significant differences between the mPPTMP195 group and the others, these findings suggest the therapeutic potential of TMP195 in fracture repair, particularly when encapsulated by mPEG-PCL (Fig. 5c).

Furthermore, histopathological assessment via staining of tissue sections revealed notable differences. HE staining indicated no significant difference in new bone area at the fracture site between the mPEG-PCL and control groups. However, both free TMP195 and mPPTMP195 groups displayed significantly larger bone scab areas. Saffron O solid green staining revealed red and green tissue formations between the upper and lower cortical bone, suggesting increased osteogenesis within the cartilage in the free TMP195 and mPPTMP195 groups. Masson’s staining demonstrated significantly larger areas of new bone scabs in mice treated with free TMP195 and mPPTMP195 compared to controls (Fig. 5b). Importantly, mice in the mPPTMP195 group exhibited more newly generated collagen fibers at bone scabs and a higher area of newly formed bone per unit tissue area compared to the free TMP195 group (Fig. 5b, e). Conversely, TRAP staining revealed significantly fewer osteoclasts in bone scabs of mice treated with mPPTMP195 nanoparticles compared to the other groups (Fig. 5b, f). These results suggest that TMP195 promotes osteoblast differentiation while inhibiting osteoclast differentiation, making it a potential fracture repair agent. Moreover, encapsulation in mPEG-PCL nanoparticles significantly enhances this reparative ability.

To delve into the molecular mechanism of this repair process, immunohistochemical staining showed no significant difference in HDAC4 and RUNX2 expression between the mPEG-PCL and control groups (Fig. 5d). However, the expression of HDAC4 was reduced after injection of free TMP195, with a more pronounced reduction observed after mPPTMP195 injection, particularly in the nucleus (Fig. 5d, g). Conversely, the expression of the osteogenic marker gene RUNX2 was upregulated in both free TMP195 and mPPTMP195 groups, with a more significant upregulation observed in the mPPTMP195 group (Fig. 5d, h). These findings suggest that mPPTMP195 promotes fracture repair more effectively than free TMP195.

### Inhibition of NRF2/HO-1 signaling pathway nuclear activation by mPPTMP195 nanoparticles via HDAC4 translocation blockade

**Fig. 6:**
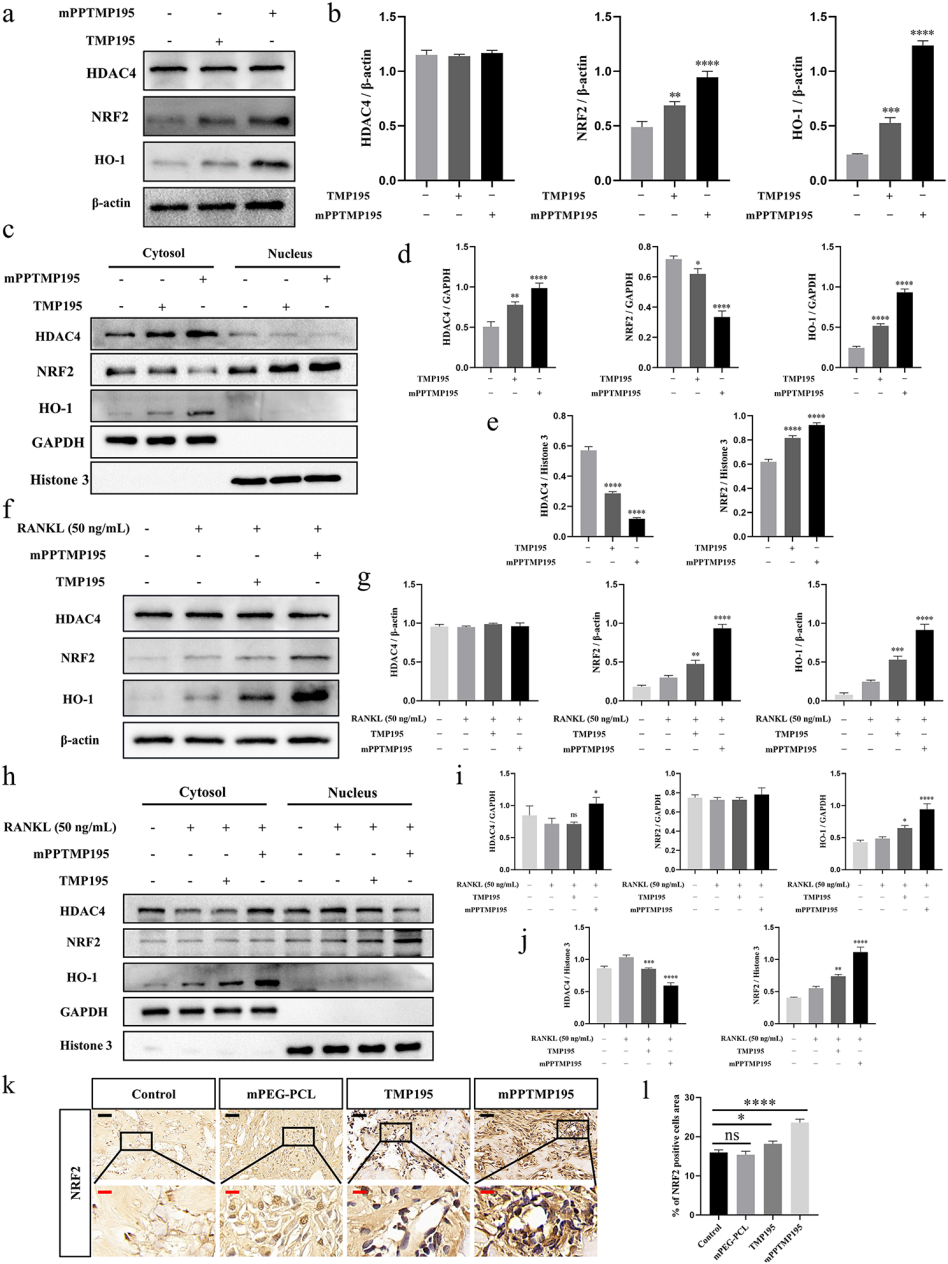
Molecular mechanism of mPPTMP195 promoting fracture repair. (**a**) Western blot analysis of HDAC4, NRF2, and HO-1 expression in BMSCs treated with mPPTMP195 for 7 days. β-actin was used as an internal reference protein. (**b**) Statistical analysis of grayscale values of total protein expression in BMSCs. (**c**) Western blot analysis of HDAC4, NRF2, and HO-1 expression in the nuclei and cytoplasmic isolates of BMSCs treated with mPPTMP195 for 7 days. GAPDH was used as a cytoplasmic endogenous reference protein, and Histone 3 was used as a nuclear endogenous reference protein. (**d**) Statistical analysis of grayscale values of cytoplasmic protein expression in BMSCs. (**e**) Statistical analysis of grayscale values of nuclear protein expression in BMSCs. (**f**) Western blot analysis of HDAC4, NRF2, and HO-1 expression in BMMs treated with mPPTMP195 for 5 days. β-actin was used as an internal reference protein. (**g**) Statistical analysis of grayscale values of total protein expression in BMMs. (**h**) Western blot analysis of HDAC4, NRF2, and HO-1 expression in the nuclei and cytoplasmic isolates of BMMs treated with mPPTMP195 for 7 days. GAPDH was used as a cytoplasmic endogenous reference protein, and Histone 3 was used as a nuclear endogenous reference protein. (**i**) Statistical analysis of grayscale values of cytoplasmic protein expression in BMMs. (**j**) Statistical analysis of grayscale values of nuclear protein expression in BMMs. (**k**) Immunohistochemical staining images of NRF2 at bone scabs of control, mPEG-PCL, TMP195, and mPPTMP195 mice. Black scale: 40 μm. Red scale: 10 μm. (**l**) Quantification of the area occupied by NRF2-positive cells. All bars are expressed as mean ± SD. ^*^P < 0.05, ^**^P < 0.01, ^***^P < 0.001 and ^****^P < 0.0001 relative to the positive control group. Differences were analyzed by Student’s t-test

To further elucidate the potential molecular mechanisms of mPPTMP195 in fracture repair, we investigated the NRF2/HO-1 signaling pathway. During BMSC differentiation into osteoblasts, we observed increased expression of NRF2 and HO-1 in total protein extracts from both the free TMP195 and mPPTMP195 groups compared to the control group, while HDAC4 expression showed no significant difference (Fig. 6a, b). Since HO-1 acts as a cytoprotective regulator of NRF2 transcription factor, TMP195 may possess antioxidant activity. Subsequently, we assessed HDAC4 and NRF2 expression by isolating cytoplasmic and nuclear extracts from BMSCs to understand their roles in osteoblast differentiation. Compared with the control group, we observed that treatment of BMSCs with free TMP195 or mPPTMP195 nanoparticles resulted in increased HDAC4 expression in the cytoplasm and decreased HDAC4 expression in the nucleus in both groups. Additionally, NRF2 expression decreased in the cytoplasm and increased in the nucleus, while HO-1 expression increased in the cytoplasm but was not detected in the nucleus. Interestingly, compared to the free TMP195 group, BMSCs in the mPPTMP195 group showed higher expression of HDAC4 and slightly lower expression of NRF2 in the cytoplasm, but the opposite trend was observed in the nucleus (Fig. 6c, d, and e). These findings suggest that both treatments inhibited the translocation of HDAC4 from the cytoplasm to the nucleus and promoted NRF2 expression in the nucleus compared with the control group, with mPPTMP195 demonstrating greater effectiveness.

Next, we similarly isolated the cytoplasmic and nuclear extracts of BMMs cells and examined the effect of the NRF2/HO-1 signaling pathway on the differentiation of BMMs into osteoblasts. We found that the expression of NRF2 and HO-1 was significantly increased in the total protein extracts of BMMs on day 5 in both the free TMP195 and mPPTMP195 groups compared with the control group, but there appeared to be no difference in the expression of HDAC4 (Fig. 6f , g). Similarly, we examined the expression of HDAC4 and NRF2 in protein extracts isolated from the nucleus and cytoplasm of BMMs, respectively. Compared with the RANKL group, there was no significant difference in the cytoplasmic expression of HDAC4, but there was a decrease in the expression of HDAC4 in the nucleus of BMMs treated with free TMP195. Additionally, there was no significant difference in the cytoplasmic expression of NRF2, which was increased in the nucleus. However, the mPPTMP195 group showed increased expression of HDAC4 in the cytoplasm and decreased expression in the nucleus compared to the RANKL group. Furthermore, there was no significant difference in the expression of NRF2 in the cytoplasm, but there was increased expression in the nucleus. In the cytoplasm, the expression of HO-1 was increased in both the free TMP195 and mPPTMP195 groups compared to the RANKL group. Additionally, mPPTMP195-treated BMMs had a more pronounced effect on eliciting differential expression of HDAC4 or NRF2 in the nucleus and cytoplasm compared to the free TMP195 group (Fig. 6h, i, and j). This suggests that mPPTMP195 inhibited the translocation of HDAC4 from the cytoplasm to the nucleus, thereby promoting the expression of NRF2 in the nucleus. Finally, immunohistochemical staining of mouse specimens revealed that the expression of NRF2 in the nucleus was enhanced in both the TMP195 and mPPTMP195 groups compared with the control group, with the enhancement being more pronounced in the mPPTMP195 group (Fig. 6e, f).

Finally, immunohistochemical staining of fractured mouse specimens showed that NRF2 expression was increased in the nuclei of cells in both the free TMP195 group and the mPPTMP195 nanoparticle group compared with the control group, and the enhancement was more pronounced in the mPPTMP195 nanoparticle group (Fig. 6k, l). These findings suggest that mPPTMP195 increased the intranuclear expression of NRF2 by inhibiting the translocation of HDAC4 from the cytoplasm to the nucleus, which led to the elevated expression of HO-1.

## Discussion

Fractures are common traumatic skeletal disorders with high clinical morbidity. Trauma is the leading cause of these disorders, with approximately 5–10% of traumas resulting in delayed or failure to heal [19]. The process of fracture repair is a complex, intricate, and continuously dynamic biological phenomenon involving the participation of multiple cellular and signaling pathways. This complex process includes cell recruitment, proliferation, and differentiation. Current clinical interventions for fractures focus on stabilizing the fracture end by external cast fixation, internal plate fixation, and closed intramedullary nail fixation. Furthermore, these treatments have inherent limitations. For instance, external fixation in a cast may not provide adequate support, internal fixation with a invasive plate may entail complications such as infection, nerve, or vascular damage, and intramedullary nailing may not offer sufficient stability for fractures located on the articular surface [20–22]. Additionally, the healing cycle of fractures is lengthy, whereas the metabolic cycle of drugs used in associated treatments is relatively short. Hence, there is an urgent need for a sustained release agent as a vehicle for drug delivery to enhance drug bioavailability.

In this study, we developed an artificial biomaterial for fracture treatment with the aim of modulating the dynamic balance between osteoclasts and osteoblasts to promote effective fracture repair. Previous efforts to address the challenges of poorly water-soluble drugs have involved various formulations, such as cyclodextrin inclusion complexes and solid lipid nanoparticle-concentrated hydrogels [23]. However, in the treatment of fractures, these approaches have not achieved the desired therapeutic effect of prolonged sustained release. Polymeric micelles are an ideal nanocarrier. These micelles represent core-shell structures formed by self-assembly of amphiphilic block polymers in an aqueous environment. The hydrophobic end forms the core while the hydrophilic end forms the shell. This structure demonstrates the ability to enhance drug solubility and bioavailability [24].

To achieve controlled release of drugs, biodegradable polymer nanoparticles are commonly used in drug delivery systems. Methoxy poly (ethylene glycol)-poly (ε-caprolactone) (mPEG-PCL) is an amphiphilic block copolymer comprising a hydrophilic fragment of PEG and a lipophilic fragment of PCL [25]. Compared to other polymers, the PCL in mPEG-PCL is biodegradable in vivo, yielding non-toxic by-products, while polyethylene glycol (PEG) enhances surface hydrophilicity, thereby reducing the likelihood of immune reactions by mitigating non-specific interactions [26–28]. Approved by the FDA, mPEG-PCL exhibits a favorable safety profile for medical applications [17, 18] and is primarily employed for drug delivery [17], drug sustained release systems [29], biomedical imaging [30], and biomaterial scaffolds [31]. In this study, we prepared mPPTMP195 nanoparticles by encapsulating TMP195 in mPEG-PCL nanoparticles to explore their potential in fracture repair. Notably, mPPTMP195 exhibited sustained release properties (Fig. 1j) and did not demonstrate cytotoxicity within the concentration range of 0–45 µg/mL (Fig. 1k, l).

ALP activity is a common indicator of the early stages of osteogenic differentiation and plays a key role in bone mineralization by initiating and promoting the formation of hydroxyapatite crystals within osteoblast matrix vesicles [32–34]. Various transcription factors, including ALP, RUNX2, Osx, and OCN, are important markers of osteoblast specificity [35, 36]. RUNX2 and Osterix are considered to be the major transcription factors controlling osteoblast differentiation and the expression of bone-related genes [37, 38], and they are expressed in mineralized tissues and osteoblasts. It is widely accepted that RUNX2 acts as a key transcription factor in bone mineralization development, inducing major osteoblast-specific genes such as ALP, OCN, and Col1α1, which are essential for osteoblast differentiation [37, 39]. Our results showed that mPPTMP195 effectively promoted early and late osteoblast differentiation by increasing ALP activity and enhancing mineralization, respectively (Fig. 2a, c). In addition, mPPTMP195 upregulated the expression of osteoblast differentiation marker genes, including ALP, RUNX2, Osx, and OCN, at the mRNA level. Furthermore, mPPTMP195 enhanced the protein expression of osteoblast marker genes, such as Col1α1, RUNX2, and Osterix (Fig. 2e, f).

RANKL is expressed by osteoblasts and bone marrow stromal cells and binds to the RANK receptor on osteoclast precursor cells to promote their differentiation into mature osteoclasts [48]. This interaction between RANKL and RANK is essential for osteoblast differentiation and contributes to the process of osteoclast formation [40–42]. Additionally, RANKL induces the expression of transcription factors, including c-Fos and NFATc1, which are essential for osteoclast differentiation [40, 43, 44]. NFATc1 is a major regulator of osteoclast formation and synergistically induces the expression of osteoclast-specific genes, such as CTSK, OSCAR, TRAP, and the calcitonin receptor, in conjunction with c-Fos [45, 46]. In our study, mPPTMP195 effectively inhibited RANKL-induced expression of c-Fos and NFATc1 in an in vitro osteoclast-induced differentiation assay, thereby suppressing the expression of osteoclast-specific marker genes and proteins (Fig. 3e , 4a, and 4b).

A series of experiments were conducted to examine the bone-repairing effect of mPPTMP195 in vivo and to study its molecular mechanisms. In a mouse femur fracture model, we observed that mPPTMP195 promoted fracture repair (Fig. 5a, b). Meanwhile, we found that mPPTMP195 inhibited HDAC4 expression in vivo, leading to increased expression of RUNX2 (Fig. 5d). We isolated cytoplasmic and nuclear proteins from mPPTMP195-treated cells to examine the distribution of HDAC4 inside and outside the nucleus. We observed that mPPTMP195 effectively blocked the translocation of HDAC4 to the nucleus during the differentiation of osteoblasts and osteoclasts, resulting in a significant accumulation of HDAC4 in the cytoplasm (Fig. 6c, h). It is well known that HDAC4 is an epigenetic modifier enzyme whose primary function is to remove acetylation modifications on histones, thereby regulating key biological processes such as gene expression and cell differentiation. Additionally, HDAC4 exhibits nucleoplasmic shuttling properties and can transmit signals from the cytoplasm to the nucleus [47–49]. Within the nucleus, HDAC4 induces histones to tightly entangle with DNA, blocking the recognition and binding of transcription factors to DNA, thereby inhibiting gene expression. Therefore, its abnormal function or absence may lead to abnormal gene expression and disease. Reduced phosphorylation of HDAC4 has been shown to result in the aggregation of HDAC4 in the nucleus, triggering the deacetylation and degradation of RUNX2, a key regulator of osteogenesis, ultimately leading to bone loss [50]. During osteoblast differentiation, inhibition of HDAC4 activates the HIF-1α/VEGFA pathway, promoting osteoblast differentiation of osteogenic precursor cells and inhibiting osteoblast activity [51]. In bone marrow mesenchymal stem cells (BMSCs), inhibition of HDAC4 expression enhances the differentiation of BMSCs into cartilage [52]. According to above, mPPTMP195 exert its bone-repairing effect by inhibiting HDAC4 and increasing RUNX2 expression, which based on blocking the translocation of HDAC4 to the nucleus.

**Scheme 1:**
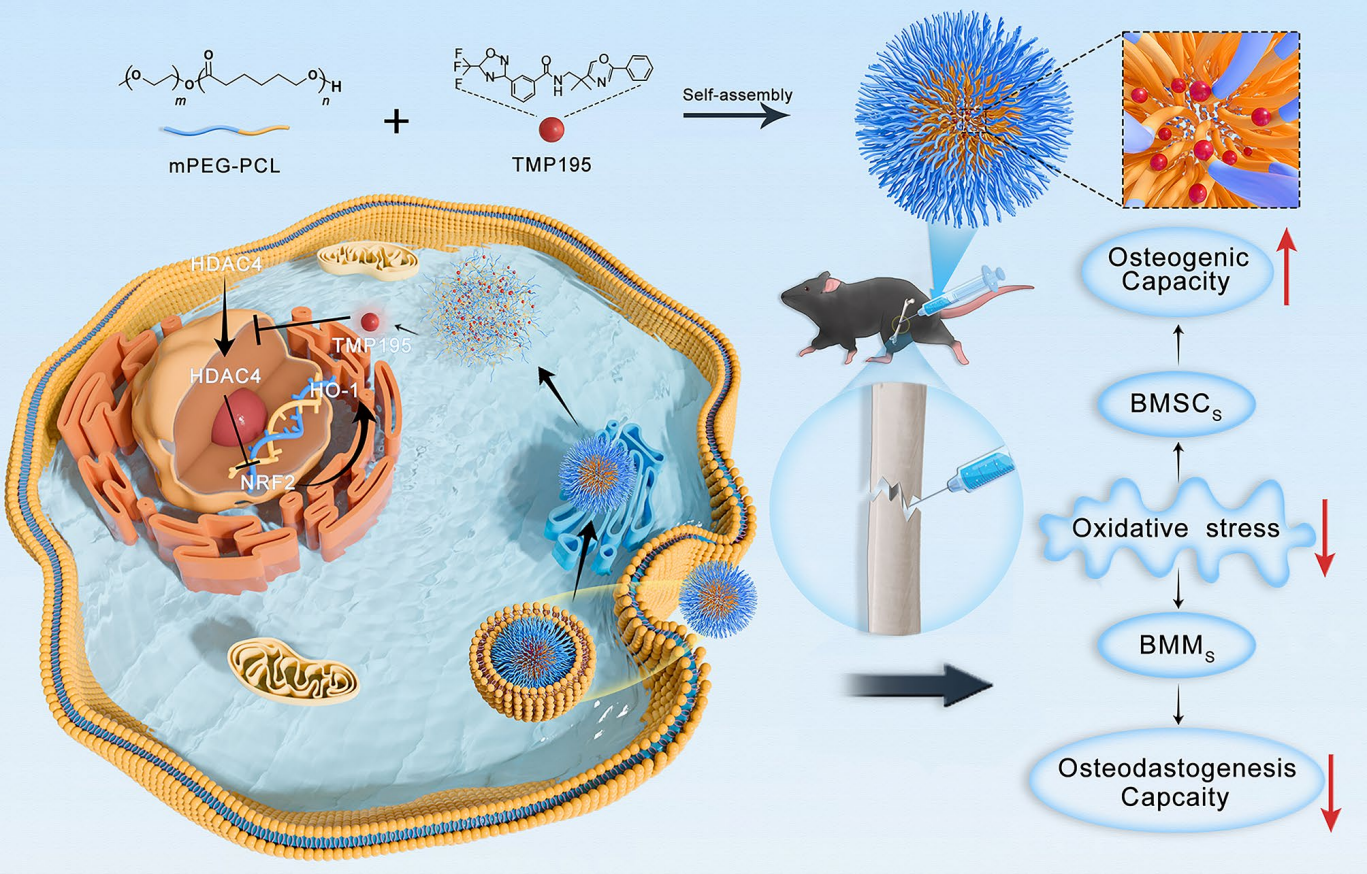
The synthesis of mPPTMP195 nanoparticles and their biological role in repairing animal fractures

The NRF2/HO-1 signaling pathway is an important regulator of cellular redox homeostasis [53], and its activation differentially affects osteoblast differentiation and osteoclast differentiation [54–58]. We aimed to elucidate whether mPPTMP195 affects this mechanism by examining NRF2 expression in the cytoplasm and nucleus. Interestingly, we found a significant increase in NRF2 expression in the nucleus of mPPTMP195-treated cells during osteoblast and osteoclast differentiation (Fig. 6c, h). Similarly, in a mouse femur fracture model, we observed that NRF2 expression was significantly higher in the mPPTMP195-treated group than in the other three groups and was more centrally localized in the nucleus (Fig. 6k). Therefore, we suggest that the NRF2/HO-1 signaling pathway may represent a downstream pathway for HDAC4. mPPTMP195 nanoparticles were released from TMP195 after cellular uptake, resulting in a reduction of HDAC4 entry into the nucleus. This interference attenuated the transcriptional repression of NRF2 by HDAC4, thereby activating the NRF2/HO-1 signaling pathway (Scheme 1). Ultimately, this molecular mechanism promotes effective fracture repair.

This study has some limitations. We recognize that systemic administration of TMP195 may have potential adverse effects on other tissues and organs. To mitigate this risk, we chose to administer the drug locally subcutaneously in the surgical model area. However, this mode of administration has the disadvantage of potential non-absorption or incomplete absorption of the drug, and repeated injections may introduce the risk of infection in the surgical area, affecting the prognosis. Thankfully, none of the animals in our study showed abnormal behavior or health problems. For future studies, we plan to delve into more comprehensive studies, including pharmacokinetics, to evaluate the blood concentrations of TMP195. It is important to emphasize that although mPEG-PCL exhibits good biosafety, its practical application needs to be considered on a case-by-case basis, taking into account factors such as the synthesis process and the physicochemical properties of the material. Rigorous biocompatibility and toxicology studies must be performed to ensure the safety and efficacy of such materials in specific applications. In addition, fracture healing is a complex and integrated process involving multiple cell types and complex interactions between osteoblasts and osteoclasts. Therefore, it remains critical to continue to explore the role of osteoblasts and osteoclasts in crust formation and bone remodeling.

## Conclusion

In conclusion, we successfully synthesized mPPTMP195 by combining mPEG-PCL and TMP195. Our results demonstrate the biocompatibility of mPPTMP195 and its significantly enhanced efficacy in fractured mice compared to free TMP195. Notably, we observed that mPPTMP195 activates the NRF2/HO-1 signaling pathway by preventing HDAC4 translocation from the cytoplasm to the nucleus. This activation promotes BMSC differentiation into osteoblasts and accelerates the fracture healing process. Therefore, mPPTMP195 emerges as a promising therapeutic strategy for fracture treatment.

## Materials and methods

### Preparation of mPPTMP195 nanoparticles

The synthetic procedure for mPEG-PCL is outlined in Fig. 1a. In a 50 mL round-bottom flask, 500 mg of mPEG-OH (Mw = 5 kDa, 0.10 mmol) and 2.28 g of ε-caprolactone (20.0 mmol) were combined with 15 mL of anhydrous CH2Cl2. Subsequently, 139 mg of TBD (1.00 mmol) was slowly added, and the resulting mixture was stirred at room temperature. After 30 min, the reaction was quenched with the addition of 1 mL of acetic acid. The resulting solution was then precipitated into an excess of diethyl ether. Following filtration, the precipitates were redissolved in CH2Cl2 and reprecipitated into an excess of diethyl ether. This dissolution–precipitation cycle was repeated three times. mPEG-PCL was obtained as a white solid (2.55 g, yield: 90%) after vacuum drying overnight at room temperature. TMP195 (purity ≥ 99.19%) was purchased from MCE (Wuhan, China). The mPEG-PCL powder and TMP195 were dissolved in DMSO, followed by the gradual addition of deionized water (10 times the volume of DMSO). The resulting solution was then dialyzed using a dialysis bag with a molecular weight cutoff of 3500 Da.

### Characterization of mPPTMP195 nanoparticles

The TMP195 solution was scanned at 200–800 nm using a UH-5300 spectrophotometer (HITACHI, Japan). A standard curve correlating absorbance at 423 nm with the concentration of TMP195 was created. In the subsequent study, the standard curve equation can be used to calculate the drug loading of TMP195 in mPEG-PCL nanoparticles and also help to determine the sustained release profile of TMP195 in mPEG-PCL. The morphology of the samples was determined by scanning electron microscopy (SEM) and transmission electron microscopy (TEM), respectively.

### In vitro release of TMP195 from nanoparticles

Dynamic dialysis was used to determine the in vitro drug release behavior of mPPTMP195 nanoparticles. The nanoparticle suspension was loaded into a pre-dialysis bag with the bottom ends of the bag clamped. The dialysis bag was placed in a conical flask containing 200 ml of PBS solution at 37.0 ± 0.5 °C and shaken gently. A sample of 4 ml was collected at specific time points and the absorbance of TMP195 was measured at 305 nm using PBS solution as a blank control. The concentration of TMP195 was calculated from the standard curve and the cumulative release rate was determined.

### Cytotoxicity assay

The cytotoxic effects of samples on cells were studied with the Cell Counting Kit-8 (CCK-8) according to the manufacturer’s protocol (Boster, China). Cells were seeded in 96-well plates at a density of 3 × 10^3^ cells/well according to the manufacturer’s instructions, and at the indicated time points, 10 µL of CCK-8 reagent was added to each well and incubated for 2 h at 37 °C in the dark. Finally, the absorbance was measured at 450 nm using an enzyme marker (Thermo Fisher Scientific, USA).

### Osteoblast differentiation culture and identification

BMSCs were isolated from the femoral head during hip arthroplasty. The bone marrow cavity was exposed and rinsed with α-modified minimal essential medium (α-MEM, Gibco) supplemented with 10% fetal bovine serum (FBS, Gibco), and 1% penicillin-streptomycin (PS, Thermo Fisher Scientific) until the marrow cavity turned pale. The cell suspension was collected in a centrifuge tube, centrifuged at 1000 rpm for 5 min, the supernatant was discarded, and the resultant cells were resuspended and cultured in a culture dish at 37 °C in a 5% CO2 environment. The resuspended cells were seeded into Petri dishes and cultured in an incubator at 37 °C in a 5% CO2 environment. The BMSCs were then inoculated into 24-well plates, and an osteogenic induction medium (Procell, China) was applied following the manufacturer’s guidelines. 20 µg/mL of TMP195 and mPPTMP195 containing 20 µg/mL of TMP195 were added, respectively, and the medium was changed every 48 h. After a 7-day induction period, alkaline phosphatase (ALP) staining was performed, and alizarin red (AR) staining was performed after 21 days to assess differentiation.

### Osteoclast differentiation culture and identification

BMMs were isolated from the tibia and femur of 8-week-old male C57BL/6 mice and cultured in α-MEM medium supplemented with 10% FBS, 1% PS, and macrophage colony-stimulating factor (M-CSF) at 37 °C in a 5% CO_2_ environment. The adherent cells in the culture were employed for subsequent experiments.

BMMs were laid flat in 96-well plates, and after adherence, 20 µg/mL of TMP195 and mPPTMP195 containing 20 µg/mL of TMP195 were added to complete α-MEM medium supplemented with M-CSF (25 ng/mL) and RANKL (50 ng/mL). The culture medium was replaced every 48 h for a duration of 6 days. Following the observation of Osteoclasts (OCs) in the control group, the cells were fixed for subsequent staining. TRAP staining was carried out utilizing a TRAP staining kit (Sigma, USA) as per the manufacturer’s instructions. The staining protocol identified TRAP-positive multinucleated cells (with three or more nuclei) as OCs. To visualize F-actin filaments, staining was performed with rhodamine-conjugated phalloidin (solarbio, China). After a 30-minute staining duration, the cells were washed with PBS and stained with DAPI (Boster, China). The staining outcomes were visualized through fluorescence microscopy (Leica, Germany).

### Quantitative RT‒PCR

BMSCs were seeded in six-well plates at a density of 1.5 × 10^5^ cells per well and incubated at 37 °C for 24 h. They were divided into the NC (negative control) group, PC (positive control) group, TMP195 group, and mPPTMP195 group. In the NC group, medium without osteoinductive components was used, while the other groups were supplemented with osteoinductive medium. The medium was changed every 3 days, and after 7 days of culture, total cellular RNA was extracted using TRIzol.

Similarly, BMMs were seeded into six-well plates at a density of 1.5 × 10^5^ cells per well and divided into the Control, RANKL, TMP195, and mPPTMP195 groups. They were incubated at 37 °C for 24 h. The Control group was incubated with α-MEM complete medium containing 25 ng/mL M-CSF (without RANKL), and the remaining groups were treated with α-MEM complete medium containing 25 ng/mL M-CSF and 50 ng/mL RANKL. The medium was changed every two days, and total RNA was extracted using TRIzol after 5 days of continuous culture.

The extracted total cellular RNA was reverse transcribed into cDNA using the PrimeScript RT Master Mix (Takara, Japan) following the manufacturer’s protocol. Quantitative polymerase chain reaction (qPCR) was performed on an Applied Biosystems QuantStudio 6 Flex Real-Time PCR System (Thermo Fisher Scientific, USA) using the TB Green PreMix Ex Taq kit (Takara, Japan). The relative expression of the target genes was normalized to the housekeeping gene 18s using the 2-ΔΔCT method. The target genes and primer sequences are detailed in Table 1.

**Table 1:** Information of the primers used for RT-PCR analysis

Gene	Froward (5’-3’)	Reverse (5’-3’)
CTSK	CACTGCCTTCCAATACGTGC	TGCATTTAGCTGCCTTTGCC
Acp-5	CAGCAGCCAAGGAGGACTAC	ACATAGCCCACACCGTTCTC
c-Fos	TTTCAACGCCGACTACGAGG	GCGCAAAAGTCCTGTGTGTT
NFATc1	CAACGCCCTGACCACCGATAG	GGCTGCCTTCCGTCTCATAGT
DC-STAMP	TTCATCCAGCATTTGGGAGT	ACAGAAGAGAGCAGGGCAAC
V-ATPase-d2	GTGAGACCTTGGAAGTCCTGAA	GAGAAATGTGCTCAGGGGCT
ALP	AACACCACCCAGGGGAAC	GGTCACAATGCCCACAGATT
Runx2	GCCTAGGCGCATTTCAGA	GCTCTTCTTACTGAGAGTGGAAGG
Osx	ATAGTGGGCAGCTAGAAGGGAGTG	ATTAGGGCAGTCGCAGGAGGAG
OCN	TCACACTCCTCGCCCTATTG	AGCCAACTCGTCACAGTCC
18s	CGGCTACCACATCCAAGGAA	GCTGGAATTACCGCGGCT

### Western blot analysis

BMSCs were seeded into six-well plates at a concentration of 5 × 10^5^ cells per well. After cell attachment, osteogenic induction medium was added. TMP195 at a concentration of 20 µg/mL and mPPTMP195 containing 20 µg/mL TMP195 were added respectively, and the medium was changed every 3 days. After 7 days of culture, total protein was extracted. Similarly, another batch of cells was cultured to extract cytoplasmic and nuclear proteins in order to assess the differences in protein expression inside and outside the nucleus during osteoblast differentiation.

BMMs were inoculated into six-well culture plates at a concentration of 5 × 10^5^ cells per well. After cell adhesion, the medium was changed to α-MEM complete medium containing 25 ng/mL M-CSF and 50 ng/mL RANKL. Control cells were treated with PBS, while experimental cells were treated with mPPTMP195 containing 20 µg/mL TMP195. The medium was renewed every two days. The cells were induced for 0, 1, 3, and 5 days, respectively, and then total protein was extracted.

Similarly, BMMs were seeded into six-well plates at a concentration of 5 × 10^5^ cells per well. Cells were allowed to attach to the surface and cultured with α-MEM complete medium supplemented with 25 ng/mL M-CSF and 50 ng/mL RANKL. TMP195 at a concentration of 20 µg/mL and mPPTMP195 containing 20 µg/mL TMP195 were added separately, and the medium was changed every two days. After 5 days of induction, total protein was extracted. Another batch of cells was cultured to extract cytoplasmic and nuclear proteins to detect changes in protein expression inside and outside the nucleus during osteoblast differentiation.

Proteins were separated by molecular size by 10% SDS-PAGE and then transferred to PVDF membranes and blocked with 5% skim milk in Tris-Buffed saline (TBS) containing 0.1% Tween-20 (TBST) for 2 h. The membranes were incubated overnight at 4 °C with the following primary antibodies, including anti-Integrin β3 (1:750, Cat.# WL04156, Wanleibio), anti-NFATc1(1:1000, Cat.# A1539, Abclonal), anti-CTSK (1:2000, Cat.# DF6614, Affinity), anti-c-Fos (1:1000, Cat.# WL03699, Wanleibio), anti-CoL1α1 (1:500, Cat.# AF7001, Affinity), anti-RUNX2 (1:1000, Cat.# CY5395, Abways), anti-Osterix (1:1000, Cat.# bs-1110R, Bioss), anti-HDAC4 (1:500, Cat.# A13510, Abclonal), anti-RUNX2 (1:1000, Cat.# AF0639, Affinity), anti-HO-1 (1:1000, Cat.# ab52947, Abcam), anti-GAPDH (1:10000, Cat.# AC001, Abclonal), anti-Histone 3 (1:5000, Cat.# A2348, Abclonal), anti-β-actin (1:1000, GB11001, Servicebio).Then, the membranes were incubated with anti-rabbit antibody (1:5000, RS0001, Immunology) for 2 h at room temperature. The immunoreactive proteins were detected with ECL. Finally, immunoreactive bands were visualized by the touch imaging system ChemiDoc™ (BIORAD, USA) according to the manufacturer’s instructions.

### Femoral fracture model

All mice were obtained from the Animal Center of Shanxi Medical University (Shanxi, China). Twenty-four 10-week-old mice were randomly divided into 4 groups of 6 mice each: the control group, the mPEG-PCL group, the TMP195 group, and the mPPTMP195 group. Surgical preparation was conducted one day before surgery, during which the hair near the knee joints of the mice was removed. Anesthesia was induced by intraperitoneal injection of 0.8% sodium pentobarbital at a dose of 70 mg/kg. The skin was sterilized with 75% alcohol. Local muscles were incised with a No. 11 scalpel blade to expose the femoral condyles. After inserting an intramedullary needle, the mouse femur was fixed to keep it level with the tabletop, fractured along the mid femur, and sutured for bandaging. Mice in the mPPTMP195 group were injected subcutaneously in the fracture area with 50 µL of mPPTMP195 containing 20 µg/mL TMP195 every other day starting on the second postoperative day for a total of four injections. Similarly, mice in the free TMP195 group were injected subcutaneously in the fracture area at the same dose as the mPPTMP195 group.

### Micro-CT scan analysis

The specimen was placed in a micro-CT machine and securely fixed to ensure stability and prevent any distortion of the layers’ morphology during the scanning process. Three-dimensional imaging of the bone was conducted under controlled scanning conditions with a scanning voltage of 70 kVp, a resolution of 10.1 μm, and an exposure time of 300 ms. A total of 300 layers were analyzed, comprising 150 layers above and below the widest diameter of the fracture line region to ensure a comprehensive evaluation. Following the scanning process, the samples were reconstructed in three dimensions and subjected to quantitative analysis for various parameters, including bone volume (BV), tissue volume (TV), bone volume fraction (BV/TV), number of trabeculae (Tb.N), thickness of trabeculae (Tb.Th), and trabecular separation (Tb.Sp).

### Histochemistry and immunohistochemistry

Mouse femur samples were fixed in 4% paraformaldehyde for 48 h and subsequently decalcified with 14% EDTA at 37 °C for 2 weeks. Sagittal sections, 5 μm thick, were prepared for staining with Hematoxylin and Eosin (H&E), Senna O Solid Green (SF), Masson’s trichrome, and anti-tartrate-resistant acid phosphatase (TRAP). Immunohistochemistry was conducted following standard protocols. Sections were incubated overnight at 4 °C with primary antibodies, including anti-HDAC4 (1:50, Cat.# D262917, Sangon Biotech), anti-RUNX2 (1:50, Cat.# AF0639, Affinity), and RUNX2 (1:50, Cat.# CY5395, Abways). Subsequently, the sections were treated with anti-rabbit secondary antibody for 1 h at 37 °C and visualized using DAB (ZSGB-BIO, Beijing, China). Finally, the areas stained with Masson’s trichrome, the number of TRAP-positive cells, and the quantification of immunopositive cells were assessed utilizing Image J.

### Statistical analysis

The statistical analysis was performed using GraphPad Prism 8 software. Comparative analysis between two groups was executed using a two independent samples t-test, while one-way analysis of variance (ANOVA) was employed for three or more groups. The data were presented as mean ± standard deviation (SD), and statistical significance was considered for P-values < 0.05, indicating the presence of statistically significant differences.

### Electronic supplementary material

Below is the link to the electronic supplementary material.

Supplementary Material 1

## Data Availability

No datasets were generated or analysed during the current study.

## Abbreviations

HDAC4: Histone deacetylase 4
RUNX2: Runt-related transcription factor 2
NRF2: Nuclear Factor erythroid 2-Related Factor 2
HO-1: Heme oxygenase-1
BMP2: Bone morphogenetic protein 2
VEGF: Vascular endothelial-derived growth factor
PDGF: Platelet-derived growth factor
BMSCs: Bone marrow mesenchymal stem cells
BMMs: Bone marrow macrophages
ALP: Alkaline phosphatase
AR: Alizarin red
Osx: Osterix
OCN: Osteocalcin
Col1α1: Collagen1α1
NFATc1: Nuclear factor of activated T cells cl
DC-STAMP: Dendritic cell-specific transmembrane protein
V-ATPase-d2: Vacuolar-type H(+)-ATPase
Acp5: Tartrate-resistant acid phosphatase
CTSK: Cathepsin K
RANKL: Receptor activator of nuclear factor-kB ligand
M-CSF: Macrophage-colony-stimulating factor
HE: Hematoxylin and eosin
TRAP: Tartrate-resistant acid phosphatase
