# Supplementary material for: mPPTMP195 nanoparticles enhance fracture recovery through HDAC4 nuclear translocation inhibition
Source: J Nanobiotechnology. 2024 May 17;22:261. doi: 10.1186/s12951-024-02436-1 (PMC11100250; doi:10.1186/s12951-024-02436-1)
Supplement: Supplementary file 1 — Supplementary Material 1 [file 12951_2024_2436_MOESM1_ESM.docx]

**
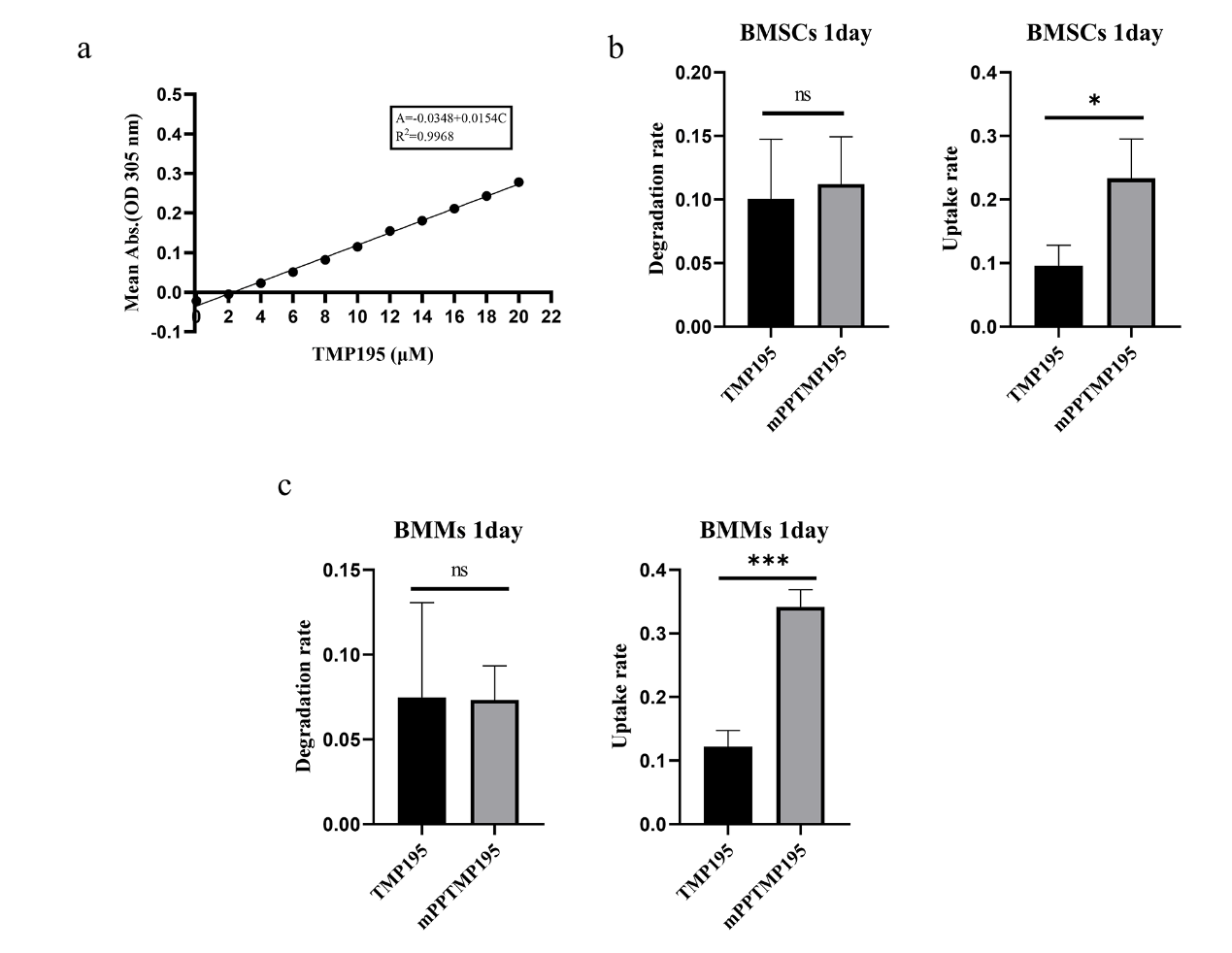
**

**Supplementary Fig. 1** (a) Standard curve of TMP195 at 305nm for different concentrations. (b) Degradation rate and uptake rate of TMP195 in BMSCs from free TMP195 and mPPTMP195. (c) Degradation rate and uptake rate of TMP195 in BMMs from free TMP195 and mPPTMP195.
